# Population attributable danger of hereditary heart breaks. Risk factors among newborns in Yazd, Iran

**Published:** 2015

**Authors:** M Taheri, A Dehghani, M Noorishadkam, SM Tabatabaei

**Affiliations:** * Faculty of Health, Department of Biostatistics & Epidemiology, Shahid Sadoughi University of Medical Sciences ,Yazd, Iran,; ** Hospital of Shahid Sadoghi, Department of Neonatal, Research Centre for premature neonate Shahid Sadoughi University of Medical Sciences, Yazd, Iran,; *** Faculty of Paramedical Science, Student Research Committee, Shahid Beheshti University of Medical Sciences, Tehran, Iran

**Keywords:** Population Attributable Risk, hereditary heart problem, Yazd, Iran

## Abstract

**Background:** hereditary heart problem are cardiac problems that develop prior to birth and influence the newborns’ basic performance of heart. Different kinds of deficiency can range from mild (e.g. a small hole between the heart chambers) to severe (like a flaw or weakness into a heart part).

**Material & Method:** This case-control research performed to evaluate PAR congenital heart defects danger parameters between newborns from 2012 to 2013 in Yazd hospitals. The defects were identified through echocardiography and recorded based on the 10th Revision of Diseases International Classification (DIC10). The total amount of cases were 96, of whom 14 were excluded regarding that the shortage of cooperation or insufficient data. One hundred and sixty five sex and region matched controls selected through random sampling involved in the research. Population Attributable Risk (PAR) utilized to evaluate and measure the priorities of the danger parameters. Finally, the Levin formula utilized to evaluate the regulate community ascribable portion.

**Result:** Regulated odds rates evaluated for the danger parameters. The outcomes revealed that the greatest odds ratio belonged to the maternal history of stillbirth, lack of multivitamin use before pregnancy, maternal obesity, and overweight. The calculation of the adjusted Population Attributable Fraction in risk factors indicated that the highest fraction of the causes of natural heart malformations was associated with overweight and obesity.

**Conclusion:** The current research showed that several factors that can impact the congenital heart defects. It concluded that although overweight and obesity had a weaker association than the other 2 parameters, they have a higher prevalence and a greater attributable risk.

## Introduction

Hereditary heart problem are cardiac problems that develop prior to birth and influence the performance and overall of the newborns’ heart. Different types of defects can range from mild (e.g. Atrial Septal Defect) to conditions [**1**].

Depending on the hereditary heart problem kind and severity, they can be asymptomatic or symptomatic discoloration of the nails, lips, tachypnea, respiratory distress, or poor feeding [**1**].

The hereditary heart problem cause in newborns is unknown; the defects happen because of genetic or chromosomal changes; they could be caused by a combination of genetic injuries and other cardiovascular hazard portions like exposure to environmental factors, maternal nutrition, and maternal drug use [**1**,**2**].

Inherent heart injuries are associated with the genetic status in 15% of the cases [**3**,**4**]. It is properly established that almost 20 to 30% of the people whom bear congenital heart diseases also suffer from physical problems [**5**,**6**]. Although a low portion of the defects are attributed to genetic issues [**3**,**4**], there is little evidence that non-genetic factors cause the defects [**7**], and no researches concentrated on the parameters affecting the disease, that is why prevention of congenital heart defects has almost been stopped due to the shortage of information on modifiable factors [**7**]. However, various studies have shown the impact of status like maternal diabetes, maternal febrile illness, congenital rubella, maternal epilepsy, folic acid, vitamin A, various drugs, age of the mother, age of the father, parental education level, history of stillbirth, maternal obesity, Turner syndrome, oral cleft, age at birth, and maternal phenylketonuria.

In USA, the illnesses are the most prevalent origin problem which claims the living of about one percent of the live births or 40,000 births each year [**2**,**8**,**9**]. The incidence of some hereditary heart problem kinds, particularly mild, has grown while the other types have remained constant, Ventricular Septal Defect being the most common case [**10**]. A study in 2002 showed that 650,000 out of 1.3 million adults lived via hereditary heart problem. In this estimation, the prevalence at parturition and the amount of the surviving instances was used [**11**].

Eighteen in every 10,000 parturition in the USA suffer from severe cardiac abnormalities [**12**]. During the years 1999 to 2006, 41,494 deaths were stated because of hereditary heart problem; nearly half of the deaths occurred before one year of age [**13**]. More than one year survival ratio in cases via myocardial injury has improved over time but its mortality is still high [**14**]. The most ratio of death in children under one month old and approximately 2.40% of the total deaths are because of hereditary heart problem in the initial 27 days of life [**15**].

In 2004, $1.4 billion were spent on congenital heart defects and nearly $511 million on the severe forms in the US, which was about 37% of all hospital costs [**16**]. In 2005, the medical care of a child with congenital heart defects cost about $100,000 with medical insurance; the costs being even higher in severe types [**17**]. Several prevalence rates of this disease have been reported worldwide [**2**,**18**,**19**], but the generally accepted estimate was about 8 in every one thousand live births [**20**]. The prevalence has increased in time, from 6 cases each 10,000 live parturition (95% CI, 4-8) during 1930 to 1934 to 9.1 cases per thousand live births after 1995. Since then, this prevalence did not change until 2011. For this reason, public health costs due to this disease are on the rise [**19**].

Among the continents, Asia has the highest and Africa has the lowest incidence with 93 (95% CI, 89-93) and 19 cases per 10,000 live births (95% CI, 11-35), respectively [**19**].

There is a clear link among the incidence of the defects and the economic status; the highest incidence rates have been reported in high-income countries (8 per thousand live births) (95%CI 7.9-8.1) [**21**]. People who suffer from this disorder require special expertise and long-term care [**22**].

Given the point of these anomalies and their impacts on the economic and psychological factors and expense of the community, the health system, and the families, and also due to their effect on the one-year mortality rate (IMR), that is a main indicator of health and community development, and since no study has investigated the factors influencing these disorders, we decided to design a study to evaluate the impact of known environmental factors on the disease and calculate the contribution of each of these factors.

## Materials and Methods

This case-control research carried to manage the attributable community danger of congenital heart defects danger portions between newborns during 2012 and 2013 in Yazd hospitals. The defects were identified through echocardiography and recorded according to the 10th Revision of International Categorization of Diseases (ICD10). The whole amount of patients was 96, of whom 14 were excluded because of the shortage of cooperation or insufficient data. One hundred and sixty five sex and region matched controls selected through random sampling involved in the research to evaluate the danger parameters. A self-administered checklist approved by experts utilized to assure the data validation. The information gathered via the records of neonates born in the hospitals, family health records in urban health databases, and contact via the neonates’ parents by collaborators working in hospitals and health care centers. Demographic properties like sex, and congenital problems including cleft lip and cleft palate were identified based on the clinical expression of the neonates admitted to the hospitals. Demographic properties of the mothers like age at conception were extracted from family health records and classified according to age classifications in similar articles (under 18, 18-35, and 36 years and upper). Paternal age at conception was concluded of family health records and classified based on the similar articles (below 40 and above 40 years). BMI evaluated via using the weight and height based on information that was recorded in the family health records (till the initial two weeks of pregnancy) and was calculated and classified based on the international classification (less than 18.5 = underweight, 24.9 to 18.5 = normal, 29.9 to 25 = overweight, more than 30 = obese) [23]. Family health records were used to evaluate social factors including occupation (housewife, employed), education (illiterate and elementary, middle school, high school diploma, associate degree, or higher), reproductive characteristics including stillbirth (yes, no), maternal medical history including diabetes according to IGT and OGTT test results (yes, no; kind 1, kind 2, and diabetes of gestational), and usage of multivitamin during pregnancy (yes, no; before pregnancy, during pregnancy). Chi-square was used to compare the predominance of danger portions between controls and cases. To evaluate if the examined risk factors had significant effects on hereditary heart problem, logistic regression was used. The factors controlling the misrepresentation of all factors entered to the logistic regression patterns. Population Attributable Risk (PAR) is one of the substantial critical determinants of state health that is closely associated with epidemiological evaluations and measures and priority risk parameters in the society were calculated. PAF is a relationship of illness in the community attributed as a danger part potentially preventable by elimination of presentation to that factor [24] and finally to calculate the adjusted Population Attributable Fraction, Levin formula given below being used.

$$PopAR\%=\frac{P_{e}(OR-1)}{P_{e}(OR-1)+1}$$

where OR indicates the odds ratio adjusted for all risk factors and Pe represents the associated prevalence risk factors in the control group.

## Results

Between 2012 and 2013, a total of 21,867 births occurred, including 96 neonates suffering from congenital heart malformations with an incidence rate of approximately 4 per 1000 births per year. About 43.9% of the newborns with these anomalies were girls and the remaining were boys.

As for registered problems, problem of ventricular septal (19.5%), patent ductus arteriosus (12%), patent ductus arteriosus + problem of atrial septal (7.3%) had the highest frequency, respectively. Moreover, 10 (12.2%) cases had oral cleft type (cleft palate, cleft lip or both). Seventeen patients died before 1 year.

The mean sympathetic and patrilineal period at the moment of perception was 28.71 ± 5.43 and 33.54 ± 5.73 years in the case team and 26.61 ± 4.76 and 30.73 ± 5.39 years in the control team. 

In order to estimate the social factors related to parents of infants by inherent malformations, the following results were obtained: 95.1% of the mothers were housewives and the rest were employed, which was almost similar to the occupational status of the mothers in the control group (91% were housewives and 9% were employed). Also, 28% of the fathers were workers, 22% were employed, and 50% were self-employed in the case group; the results were similar in the control group (23% were workers, 18.8% were employed, and 58.2% were self-employed) (**Table 1**).

**Table 1:** Distribution of congenital heart defects according to ICD-10 codes

Abnormalities according to ICD-10 code	Number	Percent
Q21 ( Problem of ventricular septal)	16	19.5
Q21/ Q25.6 (ventricular septal defect/ Stenosis of Pulmonary Artery)	3	3.7
Q21/ Q25 (ventricular septal defect/ Patent Ductus Arteriosus)	3	3.7
Q25 ( control ductus arteriosus )	10	12.2
Q25.1 (Coarctation of Aorta)	4	4.9
Q23 (Congenital Stenosis of Aortic Valve)	5	6.1
Q21.1 (Atrial Septal Defect)	9	11
Q24.9 (Congenital malformation of Heart, Unspecified)	5	6.1
Q25.6 (Stenosis of Pulmonary Artery)	4	4.9
Q24.9/ Q21 (patent ductus arteriosus/ atrial septal defect)	3	3.7
Q25/ Q21.1 (patent ductus anteriosus/ Atrial Septal Defect)	6	7.3
Q21.3 ( Fallot Tetralogy)	3	3.7
Q21.1/ Q25 / Q21( Atrial Septal Defect/ patent ductus arteriosus/ ventricular septal defect)	1	1.2
Q21.2/ Q21/ Q25 (Atrioventricular Septal Defect/ Atrial Septal Defect)	1	1.2
Q21/ Q21.1 (Atrial Septal Defect/ patent ductus arteriosus)	2	2.4
Q25/ Q25.1/ Q21.1 (ventricular septal defect/ Coarctation of Aorta/ patent ductus arteriosus)	1	1.2
Q21/ Q22.1 (Atrial Septal Defect/ Congenital Pulmonary Valve Stenosis)	1	1.2
Q21/ Q25/ Q22 (Atrial Septal Defect/ Ventricular Septal Defect/Pulmonary Valve Atresia)	1	1.2
Q24.5 (Malformation of Coronary Vessels)	1	1.2
Q22.1/ Q21.1 (Congenital Pulmonary Valve Stenosis/ Patent ductus arteriosus)	1	1.2
Q25/ Q25.6 (ventricular septal defect/ Stenosis of Pulmonary Artery)	1	1.2
Q21.2/ Q22.4 (Atrioventricular Septal defect/ Congenital Tricuspid Stenosis)	1	1.2
Total	82	100

In the case group, 37.1% of the parents had a cesarean part and 62.2% had a natural vaginal birth versus 71.5% a natural vaginal birth and 28.5% a caesarean section in the control team.

It stated that 13.5 percent of the mothers in the case group and 8.5% of the mothers in the control team used medications over the pregnancy.

According to the findings of the univariate logistic regression, the odds rate of the danger parameters calculated, which indicated that the highest proportions were a stillbirth history in mothers, maternal obesity, lack of multivitamin use before pregnancy, and maternal age at conception (**Table 2** and **3**).

**Table 2:** Risk factors

Risk factors	Exposure	N (%)		Total (%)	Chi square	P value
		Case	Control			
History of stillbirth in women	No	76 (92.7)	163 (98.8)	239 (96.8)	6.514	0.011
	Yes	6 (7.3)	2 (1.2)	8 (3.2)		
Maternal Diabetes	No	74 (90.2)	159 (96.4)	233 (94.3)	3.837	0.05
	Yes	8 (9.8)	6 (3.6)	14 (5.7)		
Taking multivitamins before pregnancy	No	12 (14.6)	6 (3.6)	18 (7.3)	9.806	0.002
	Yes	70 (85.4)	159 (96.4)	229 (92.7)		
Body Mass Index	18>	3 (3.7)	13 (7.9)	16 (6.5)	15.89	0.001
	18-24.9	28 (34.1)	88 (53.3)	116 (47)		
	25-29.9	32 (39)	50 (30.3)	84 (30.4)		
	30<	19 (23.3)	14 (8.5)	31 (12.6)		
Mother’s age (over 35 years)	NO	72 (87.8)	160 (97)	232 (93.9)	8.066	0.005
	Yes	10 (12)	5 (3)	15 (6.1)		
Father’s age (over 40 years)	No	73 (89)	159 (96.4)	232 (93.9)	5.173	0.028
	Yes	9 (11)	6 (3.6)	15 (6.1)		

**Table 3:** Crude odds ratio of the danger factors of hereditary heart problem

Risk Factors	Odds Ratio	95% CI	P Value
History of stillbirth in mother	6.34	1.269-32.620	0.025
Maternal Diabetes	2.865	.960-8.554	0.059
Not taking multivitamins before pregnancy	4.543	1.639-12.592	0.004
Overweight	2.011	1.088-3.719	0.26
Obesity	4.625	1.869-9.569	0.006
Mother’s age at conception (over 35 years)	4.444	1.466-13.472	0.008
Father’s age at conception (over 40 years)	3.267	1.121-9.520	0.030

Then, the adjusted odds ratios were calculated for risk factors. The results showed that the highest odds ratios belonged to a stillbirth history in mothers, lack of multivitamin use before pregnancy, parental obesity and overweigh. The calculation of adjusted Population Attributable Fraction of the danger factors showed that the highest proportion of the origins of innate heart abnormalities were linked with overweight and obesity (**Table 4**).

**Table 4:** Adjusted odds ratio and population attributable risk fraction of congenital heart defects

Risk Factors	Odds Ratio	95% CI	P value	PAF%*
History of stillbirth in mother	7.846	1.242-49.563	0.028	8.5
Maternal Diabetes	1.978	.531-1.649	0.281	-
Not Taking multivitamin prior pregnancy	4.381	1.468-13.077	0.008	7.6
Overweight	2.091	1.094-3.994	0.026	19.7
Obesity	3.015	1.234-7.365	0.015	24.8
Mother’s age at conception (over 35 years)	3.084	.822-11.562	0.095	-
Father’s age at conception (over 40 years)	.061	.13-2.791	0.516	-
*Population Attributable Fraction				

## Discussion

In the current research, the risk factors reported in other studies were assessed and our results were compared with their findings. Several danger parameters are simultaneously examined and the adjusted population attributable risk index was calculated.

The results indicated that the average age of the mothers was more in the case team than the control one. Then, after categorizing the mothers into two groups, 30 years and younger, clear variations seen among the cases and the controls. Consistent with these results, other researches, like a research performed via Jenitta Reefhuis et al. in 2000 (OR=1.12, 95%CI 1.03-1.22) [**25**] and another study performed by Kathy J. Jenkins et al. in 2007 (OR=1.7, 95%CI 1.1-2.7) reported similar results. These findings proposed that the maternal age was a potential risk factor for a child with congenital heart defects [**7**].

In our study, the majority of the mothers were housewives in controls and cases. A research performed by Lynnk Cary et al. in 2002 showed that the mothers majority in controls and cases and were employed (about 63.3% and 83.3% of the cases and controls respectively). Generally, occupation did not appear to be an essential risk factor.

Although the parental stillbirth history estimated in a few studies, it is one of the parameters examined in our study and the results indicated a relatively strong association between this risk factor and the disease (OR=6.43, P=0.025). Moreover, a research performed via Kathy J. Jenkins et al. in 2007 indicted that a stillbirth history in mothers may be a danger parameter for subsequent abnormal childbirth (OR=5.61, 95 percent CI 1.94-16.2) [**7**].

The next evaluated risk factor was the lack of the application of multivitamin appendices including acid of folic before pregnancy. It was seen that the odds ratio of the shortage of multivitamin use before pregnancy had a clear link with giving birth to a child with heart defects (OR=4.54, p=0.004). As we sought to calculate the adjusted population attributable risk, this factor was considered as “lack of use” against mothers who used multivitamin supplements involving acid of folic. Several studies assessed the effect of multivitamin application and realized that geting multivitamins could be a protective factor against the danger of hereditary heart problem. For example, a research performed in 2009 by Raluca LonsecuIttu et al. showed that the fortification of agricultural products with folic acid significantly reduced the danger of hereditary heart problem (RR=0.94 95%CI 0.90-0.97) [**26**].

The study performed in 2007 by Kathy J. Jenkins et al. showed that taking folic acid would prevent hereditary heart problem (RR=0.42, 95%CI 0.319-0.98) [**7**]. Another study by Lorenzo D. Botto in 2000 found that taking multivitamin supplements could help prevent congenital heart defects [**27**].

The reason for not using multivitamin before pregnancy in most of the mothers in the current research was the shortage of data on its benefits or unintended pregnancy.

Overweight and obesity was an important risk factor in most non-infectious diseases, and this defect was not an exception. In this research, overweight and obese mothers in the initial two weeks of pregnancy were identified by using the International Classification of BMI, which indicated a clear link among overweight and obesity and birth problems. The intensity of association was calculated for overweight (OR=2.01, P=0.26) and obesity (OR=4.62, P=0.000). Consistent with these results, a meta-analysis conducted in 2009 by Katherine J. Stothard showed that maternal obesity had a significant relationship with heart problems in Childs (OR=1.2, 95%CI 1.09-1.3) [**27**]. A research performed in 2002 by Margaret L. Watkins furthers indicated that overweight (OR=2, 95%CI 1-3.8) and obesity (OR=2,95%CI 1.2-3.1) were the risk factors of heart defects in children [**28**].

A research performed in 2002 by Marie I. Cedergen confirmed our results; in the current research, overweight (OR=1.18, 95%CI 1.09-1.27) and obesity (OR=1.41, 95%CI 1.22-1.64) were specified as danger parameters for this disease [**29**].

The results of a research by Janes L Millls in 2001 were in line with these results, as she reported overweight (OR=1.15, 95%CI 1.07-1.23) was a danger parameter for hereditary heart problem [**30**].

It seemed that diabetes also served as a danger parameter for hereditary heart problem. Our study showed that maternal diabetes can leads to the child birth via heart problem. The reason why this finding was not analytically clear can be the small amount of the subjects (OR=2.86, P=0.059). However, other studies showed a clear link among the cardiac defects and maternal diabetes. A research performed in 1992 by Ramos Attroyo showed a link among hereditary heart problem and insulin-dependent diabetes (OR=5.5, 95%CI 1.2-24.8), diabetes type 2 (OR=2.9, 95%CI 1.2-7.2), and diabetes of gestational (OR=1.9, 95%CI 1.1-3.4) in moms [**31**].

A survey conducted via Becerra JE et al. in 1990 showed diabetes mellitus as a danger parameter for heart problem (RR=20.6, 95%CI 2.5-168.5) [**32**].

The mean paternal age is associated with hereditary heart problem; hence, we assessed this factor and our hypothesis was accepted (OR=3.267, p=0.030). Consistent with this finding, a study conducted in 2000 by Bassili et al. showed that paternal age above 40 was linked with hereditary heart problem (OR=2.7, 95%CI 1.5-4.85) [**33**].

The above-mentioned findings got from the crude odds ratios. After this analysis, all variables entered to the logistic regression pattern and the adjusted odds ratios were calculated with the Enter method. Four risk factors from the above-mentioned variables including obesity, overweight, lack of the multivitamin supplements usage involving acid of folic, and a stillbirth history were statistically significant. The results suggested that other factors might exist due to the confounding factors or the impact that these factors had on each other.

The calculation of the community attributable risk, which is an indicator of public health closely related with epidemiology, indicated that four factors together are in charge of 60.6% of the hereditary heart problem in the community. The highest population attributable risk belonged to obesity, (24.8%), overweight (19.7%), stillbirth history (8.5%), and application shortage of multivitamin supplements involving acid of folic (7.6%). Since PAR is linked with the relationship intensity among risk factors and the findings as well as the risk factors prevalence, it is seen that although obesity and overweight linked via a weaker intensity than the other two factors, they had a higher prevalence and higher attributable risk.

It must be considered that the attributable risk is a theoretical concept, mostly used for planning and prioritizing preventive interventions. In practice, risk factors can never be eliminated in the society. It is not possible to eliminate the impact of one factor while the other factors are kept constant.

## Conclusions

This study showed that several factors could affect hereditary heart problem. Odds ratio is an indicator that demonstrates the association strength among outcome and exposure when the risk factor prevalence has a significant and positive impact on the PAR.
